# Perceived impact of patients’ suicide and serious suicidal attempts on their treating psychiatrists and trainees: a national cross-sectional study in Saudi Arabia

**DOI:** 10.1186/s12888-023-05042-x

**Published:** 2023-08-18

**Authors:** Maha Alshutwi, Moayad Alawad, Mohammed Alammari, Mohannad Almanea, Rayan Alhumaid, Azzam S. Alkhalifah, Fahad D. Alosaimi

**Affiliations:** 1 Saudi Commission for Health Specialties, Riyadh, Saudi Arabia; 2 College of Medicine, Qassim University, Buraydah, Saudi Arabia; 3 King Fahad Specialist Hospital, Buraydah, Saudi Arabia; 4 Department of Psychiatry, College of Medicine, King Saud University, Riyadh, Saudi Arabia

**Keywords:** Suicide, Patient, Psychiatrist, Prevalence, Impact, Trauma

## Abstract

**Background:**

Patient suicides are significant events that tremendously affect psychiatrists— personally and professionally. Very few studies have focused on studying the impact of both serious suicidal attempts and completed suicide on psychiatrists and psychiatry trainees.

**Aim:**

This study assessed the prevalence and impact of patient suicide and serious suicidal attempts on psychiatrists and psychiatry trainees in Saudi Arabia.

**Methods:**

This national cross-sectional study of psychiatrists and psychiatry trainees was conducted in Saudi Arabia. Participants completed an online self-administered questionnaire to assess emotional and professional impacts and the traumatic impact of patient suicide using the Impact of Event Scale-Revised (IES-R).

**Results:**

178 psychiatrists were enrolled in this study. The prevalence rate of patient suicide among participants was 38.8%, and they experienced adverse emotional reactions. Additionally, among those who were not exposed to patient suicide, 12.9% reported exposure to serious suicide attempts, and almost all of them experienced related negative emotions. The most frequently reported emotions were sadness (61.95%), shock (48.91%), and guilt (25%), and these emotions lasted longer in completed patient suicide cases than attempted suicide. Nearly 84% of participants who experienced suicide reported its impact on their profession. The most reported professional impacts were increased focus on suicide cues, attention to legal aspects, and a tendency to hospitalize. Of participants who experienced suicide, 75.4% reported that the overall impact of suicidal events on their professional practice had improved. Of the total number of respondents who experienced either suicide or serious suicidal attempts, 10.9% reported symptoms of PTSD.

**Conclusions:**

The study highlighted the emotional and professional burden that psychiatrists and psychiatry trainees experience due to patient’s completed suicides and serious suicidal attempts. Additionally, it emphasized the need for further research to study the benefits of implementing preparatory and training programs to help trainees and psychiatrists in such instances.

## Introduction

Suicide is defined as the act of killing oneself intentionally [1]. It is a leading cause of death worldwide, with up to 703,000 people dying of suicide annually [2]. According to WHO, the suicide rate in 2019 was six individuals per 100,000 people in Saudi Arabia [3]. The percentage of people exposed to suicide by a family member, friend, or acquaintance in their lifetime is approximately 21% [4]. This exposure is linked to an increased risk of adverse psychological and physical effects [5, 6]. One of the consequences of suicide is suicide bereavement which has significant adverse educational and functional outcomes [7]. Furthermore, previous exposure to suicide is an acknowledged risk factor for suicide attempts [8]. Mental health practitioners are among those exposed to suicide, with a rate of 31–69%[9]. Patient suicide can influence psychiatrists’ professional lives, emotional well-being (e.g., shock, sadness, self-blame, guilt, anxiety, and loss of confidence), and performance, which may lead to severe acute stress disorder and post-traumatic stress disorder (PTSD) symptoms [10–12]. A study reported that psychiatrists have higher levels of work-related emotional exhaustion and severe depression compared to other disciplines [13].

Studies have looked into the factors contributing to variabilities in impact, such as length of professional practice [14, 15], gender [16–18], and relationship with the patient [19, 20].

Consultants and psychiatry trainees differ in emotional responses to patient suicide and suicide attempts. For example, psychological pain, guilt, and self-doubt are more likely to be undergone by trainees rather than consultants when experiencing patient suicidal attempts. In contrast, embarrassment and self-doubt were more common among consultants whose patients committed suicide. The effect of completed patient suicide is likely more severe than that of attempted suicide. For instance, shock following a completed patient suicide was more common than after an attempted suicide for trainees and consultants [21].

Only a few studies have concentrated on the emotional impact of a patient’s suicide attempt, even though such behaviors are more common than completed patient suicide. Therefore, there is a need to study the impact of both completed patient suicide and suicide attempts.

The study is critical, specifically in Saudi Arabia, where cultural factors may shape reactions and responses to patient suicide. The number of studies looking into suicide in the Arab world is small, with many highlighting the low acceptability of the subject [22–25].

Mental health care in Saudi Arabia has gone through many advances in the past 30 years; however, there remain areas of improvement, including the number of psychiatrists, which is low compared to the average number around the world [26]. Suggesting that psychiatrists have overloaded schedules and are possibly exposed to more patients.

The findings of this study aspire to provide valuable insights that can inform future policies, training programs, and support mechanisms for psychiatric staff members and trainees, aiming to improve their well-being and enhance patient care.

The goals of our study were to identify the prevalence of patient suicides and serious suicidal attempts experienced by psychiatrists and psychiatry trainees in Saudi Arabia and to assess its impact on their personal and professional lives.

## Methodology

All psychiatrists registered at the Saudi Commission for Health Specialties (SCHS), a scientific professional body tasked with supervising and certifying all healthcare workers and medical trainees in Saudi Arabia, were approached to participate in the study.

### Study design

This cross-sectional study was conducted between March and August 2020. This study was approved by the Institutional Review Board of the College of Medicine of King Saud University in Riyadh, Saudi Arabia.

### Population

Participants were practicing psychiatrists and psychiatry trainees from across Saudi Arabia. A psychiatry consultant is a physician who is board certified in psychiatry and has practiced for at least three years [27].

### Recruitment

As a low response rate was expected based on rates from previous similar local studies (5.1% among physicians and 25% among residents) [28, 29], we contacted all psychiatrists and trainees registered at SCHS to reach the desired sample size. We sent three consecutive emails to all psychiatrists registered at the SCHS, in which we explained the study aims and study questionnaire and attached the informed consent form. Of the 1170 registered psychiatrists/trainees at the SCHS, 137 responded with completed forms. Following this, the questionnaire was again sent through official working social platforms, and we received 55 additional responses. We obtained 192 completed forms, of which 14 were excluded owing to missing information. Any participant who did not encounter a patient suicide was asked about a serious suicide attempt. Any participant who did not encounter a serious suicide event was asked about different coping mechanisms towards patients’ suicide and suicidal behaviors. A serious suicide event is defined as an attempt that leads to permanent damage or disability (e.g., facial disfigurement, fractures).

### Data collection tool

We developed an online, self-administered questionnaire (available upon request) based on a literature review. Then, a multi-disciplinary committee covering psychiatry, academia, and epidemiology validated the questionnaire’s content. After that, the questionnaire was piloted by participants (N = 20) and modified based on the feedback before widespread distribution.

The electronic questionnaire started by inquiring about participants’ socio-demographic information, then asked if the participants had experienced a patient dying by suicide; if participants answered affirmatively, they were then asked further information:(suicide frequency, the patient’s characteristics, and the impact of the most distressing patient suicide experience in terms of emotional reactions, impact on professional life and coping mechanisms). They were also requested to fill out the Impact of Event Scale-Revised (IES-R) to assess for traumatic impact. If participants answered no, then they were asked about experiencing a patient’s serious suicide attempt; if they answered yes to this question, then they were requested to answer the same questions that participants who experienced a patient dying by suicide answered. If they also answered no, they were asked about overall coping mechanisms concerning patient suicide and suicidal behaviors. (Results of common coping strategies and habits that psychiatrists/trainees develop following patient suicide/attempt have been reported in another manuscript submitted for publication).

The Impact of Event Scale-Revised (IES-R) was used to assess the traumatic impact on the participants [30]. The IES-R comprises 22 items across 3 domains (8 avoidance items, 8 intrusion items, and 6 hyperarousal items). The severity of symptoms was evaluated on a 5-point scale (0 to 4) for the previous one-week period of the event. A scoring range of 0 to 88 was used, with a cut-off of ≥ 25 [30] for clinical concern PTSD.

### Data management and analysis plan

Statistical analyses were performed using Statistical Packages for Software Sciences (SPSS) version 26 (Armonk, New York, USA). Data were presented as numbers (percentages) for all qualitative variables, while median, minimum, maximum, and mean ± standard deviation were used to present all quantitative variables. Between comparisons, the chi-square test and Fisher’s exact test were used for categorical variables, whereas the Kruskal Wallis test and Mann-Whitney U test were used for continuous variables. Normality tests were performed using the Shapiro-Wilk test. Intrusion, avoidance, and hyperarousal scores followed an abnormal distribution. Therefore, nonparametric tests were performed. A P-value < 0.05 was considered the significant level for all statistical tests.

## Results

### Demographics

178 participants (response rate = 19.1%) were enrolled, and we evaluated their reactions to patient suicide or serious suicide attempts.

Table 1 shows participants’ socio-demographic characteristics. 93 (52.2%) were in the younger age group (< 40 years); 127 (71.3%) of them were men, and 51 (28.7%) were women. The majority were Saudi 110 (61.8%). Psychiatrists (62 consultants and 63 specialists) accounted for 121 (68.0%), and residents (46 trainees and seven service residents) were 57 (32%). Nearly half of the participants had 10 years or less of practice 90 (50.6%). Adult psychiatry 43 (24.2%) was the most common subspecialty of psychiatrists.

**Table 1:** Socio-Demographic information of participants concerning suicidal event

Study Variables	Overall N (%) ^(n=178)^	Witnessed Suicidal event			P-value ^§^
		**Died by suicide** **N (%)** ^**(n=69)**^	**Attempted** **N (%)** ^**(n=23)**^	**No** **N (%)** ^**(n=86)**^	
**Age group**					
< 40 years	93 (52.2%)	24 (34.8%)	15 (65.2%)	54 (62.8%)	0.001 **
≥ 40 years	85 (47.8%)	45 (65.2%)	08 (34.8%)	32 (37.2%)	
**Gender**					
Male	127 (71.3%)	55 (79.7%)	17 (73.9%)	55 (64.0%)	0.094
Female	51 (28.7%)	14 (20.3%)	06 (26.1%)	31 (36.0%)	
**Nationality**					
Saudi	110 (61.8%)	32 (46.4%)	17 (73.9%)	61 (70.9%)	0.003 **
Non-Saudi	68 (38.2%)	37 (53.6%)	06 (26.1%)	25 (29.1%)	
**Position**					
Residents	57 (32.0%)	11 (15.9%)	09 (39.1%)	37 (43.0%)	0.001 **
Psychiatrists	121 (68.0%)	58 (84.1%)	14 (60.9%)	49 (57.0%)	
**Length of practice**					
≤ 10 years	90 (50.6%)	20 (29.0%)	16 (69.6%)	54 (62.8%)	< 0.001 **
> 10 years	88 (49.4%)	49 (41.0%)	07 (30.4%)	32 (37.2%)	
**Psychiatrist subspecialty**					
Child and adolescent	06 (03.4%)	04 (05.8%)	0	02 (02.3%)	0.006 **
Adult psychiatrist	43 (24.2%)	24 (34.8%)	03 (13.0%)	16 (18.6%)	
Geriatric psychiatrist	03 (01.7%)	02 (02.9%)	01 (04.3%)	0	
Psychosomatic medicine	08 (04.5%)	02 (02.9%)	01 (04.3%)	05 (05.8%)	
Addiction	11 (06.2%)	07 (10.1%)	01 (04.3%)	03 (03.5%)	
None	101 (56.7%)	25 (36.2%)	17 (73.9%)	59 (68.6%)	
Others	06 (03.4%)	05 (07.2%)	0	01 (01.2%)	
**Place of work**					
Central region	14 (07.9%)	05 (07.2%)	01 (04.3%)	08 (09.3%)	0.351
Eastern region	23 (12.9%)	14 (20.3%)	02 (08.7%)	07 (08.1%)	
Western region	56 (31.5%)	16 (23.2%)	09 (39.1%)	31 (36.0%)	
Southern region	25 (14.0%)	12 (17.4%)	03 (13.0%)	10 (11.6%)	
Northern region	60 (33.7%)	22 (31.9%)	08 (34.8%)	30 (34.9%)	
**Work sector**					
Government	149 (83.7%)	56 (81.2%)	20 (87.0%)	73 (84.9%)	0.791
Private	11 (06.2%)	05 (07.2%)	02 (08.7%)	04 (04.7%)	
Both	18 (10.1%)	08 (11.6%)	01 (04.3%)	09 (10.5%)	

^§^P-value has been calculated using Chi-square test

** Significant at p < 0.05 level

### Prevalence

Among the enrolled participants, 69 (38.8%) experienced patients who died by suicide, 23 (12.9%) had patients who had a serious suicide attempt, and the remaining 86 (48.3%) did not witness any suicide events. As reported by participants, the average number, whether inside or outside Saudi Arabia, was 3.3, with a median of 2. However, in Saudi Arabia, only the average number of suicide cases per participant was 2.06 (It is worth noting that 38.2% of the participants were non-Saudis). The prevalence of those who experienced patients dying by suicide was more common among the older age group (≥ 40 years) (p = 0.001), psychiatrists (p = 0.001), and those working in the government sector. In comparison, the prevalence of those who experienced a patient’s serious attempted suicide was among younger age groups (< 40 years) and those with 10 years or less experience (p < 0.001).

### Patients’ characteristics

Table 2 the characteristics of patients who died by suicide and attempted suicide as witnessed by psychiatrists and trainees. Following the results, the prevalence of attempted suicide was statistically significantly higher among Saudis (p = 0.040), those who were seen by the psychiatrist more than 3 days past (p = 0.015), those who had the last consultation more than 4 days past (p = 0.004) and those who attempted suicide by means of jumping or precipitation (p = 0.002).

**Table 2:** Characteristics of patients who died by suicide and attempted suicide as witnessed by psychiatrists and trainees

Characteristics	Committed N (%) ^(n=69)^	Attempted N (%) ^(n=23)^	P-value ^§^
**Patient age group**			
≤ 35 years	44 (63.8%)	16 (69.6%)	0.801
> 35 years	25 (36.2%)	07 (30.4%)	
**Gender**			
Male	51 (73.9%)	13 (56.5%)	0.126
Female	18 (26.1%)	10 (43.5%)	
**Patient status**			
Outpatient	36 (52.2%)	09 (39.1%)	0.064
Inpatient	28 (40.6%)	08 (34.8%)	
ER	05 (07.2%)	06 (26.1%)	
**Nationality**			
Saudi	44 (63.8%)	20 (87.0%)	0.040 **
Non-Saudi	25 (36.2%)	03 (13.0%)	
**History of previous attempts**			
Yes	26 (37.7%)	14 (60.9%)	0.088
No/I don’t know	43 (62.3%)	09 (39.1%)	
**Duration to have known the patient (months)**			
≤ 3 months	36 (52.2%)	12 (52.2%)	1.000
> 3 months	33 (47.8%)	11 (47.8%)	
**Last time seeing the patient (days)**			
≤ 3 days ago	43 (62.3%)	07 (30.4%)	0.015 **
> 3 days ago	26 (37.7%)	16 (69.6%)	
**Last consultation (days)**			
≤ 4 days ago	40 (58.0%)	05 (21.7%)	0.004 **
> 4 days ago	29 (42.0%)	18 (78.3%)	
**Duration of emotional reaction**			
≤ 3 weeks	28 (40.6%)	16 (69.6%)	< 0.001 **
> 3 weeks	08 (11.6%)	07 (30.4%)	
Unknown	33 (47.8%)	0	
**Method of suicide**			
Hanging	23 (33.3%)	01 (04.3%)	0.002 **
Jumping/precipitation	14 (20.3%)	10 (43.5%)	
Medication overdose/poisoning	10 (14.5%)	02 (08.7%)	
Fire weapon	07 (10.1%)	01 (04.3%)	
Cutting/phlebotomy	05 (07.2%)	05 (21.7%)	
Drowning	01 (01.4%)	01 (04.3%)	
I don’t know	01 (01.4%)	03 (13.0%)	
Others	08 (11.6%)	0	
**Quality of relationship with patients**			
Similar to relationships with other patients	54 (78.3%)	20 (87.0%)	0.119
Closer than with other patients	13 (18.8%)	01 (04.3%)	
More hostile than with other patients	0	01 (04.3%)	
Others	02 (02.9%)	01 (04.3%)	
**Suicide risk prediction**			
Yes	55 (79.7%)	18 (78.3%)	1.000
No/I don’t know	14 (20.3%)	05 (21.7%)	

^§^ P-value has been calculated using Fischer Exact test

** Significant at p < 0.05 level

### Completed patient suicide

**Fig. 1:**
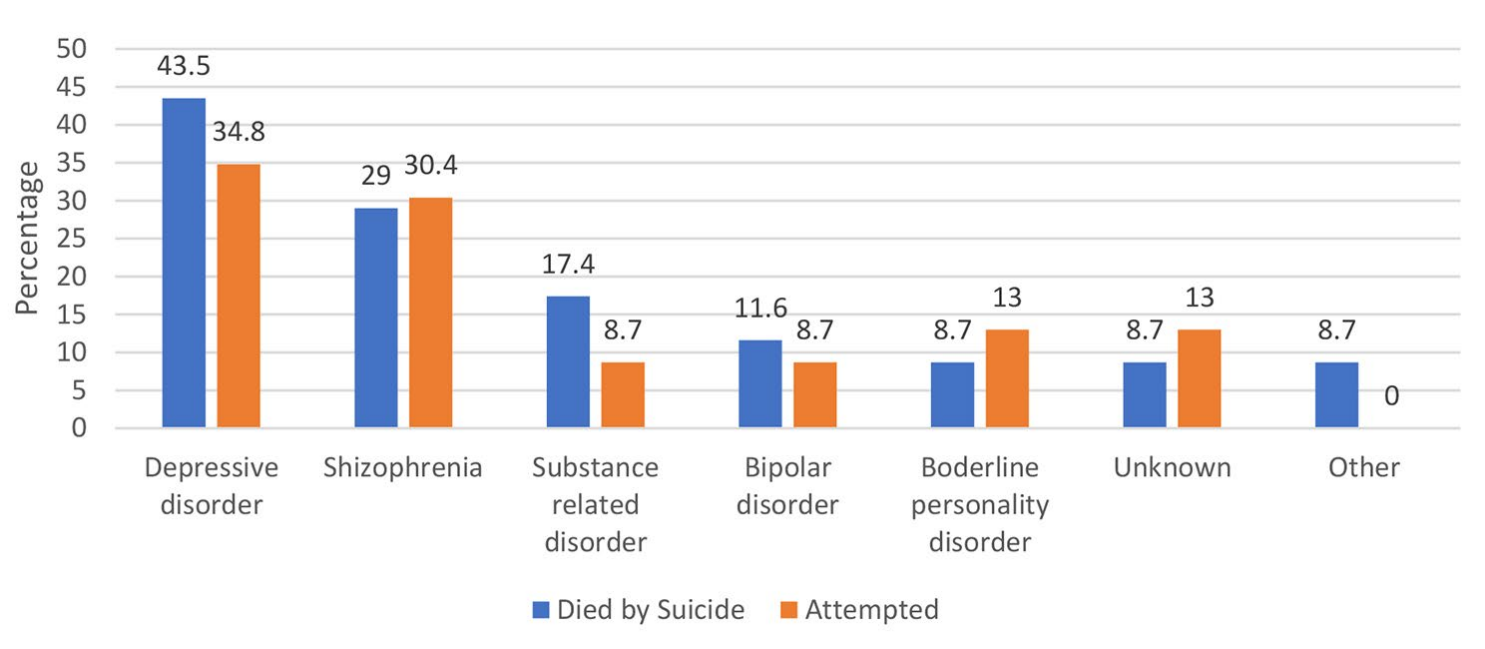
Diagnosis distribution between patients who died by suicide and attempted suicide

Of the 69 patients who died by suicide, 51 (73.6%) were men, while 18 (26.1%) were women. Most were aged 35 years or less (63.8%). Most were Saudis 44 (63.8%). The most common suicide methods were hanging (33.3%), jumping/precipitation (20.3%), and medication overdose/poisoning (14.5%). Many psychiatrists knew their patients for three months or less (52.2%), while some (47.8%) knew their patients for over three months. In Fig. 1 we demonstrate the diagnoses of the patients who died by suicide. The time of the last consultation was more than one week to one month before death in the majority of responders (23.2%). To the structured question, “What was the quality of your relationship with the patient,“ 78.3% of psychiatrists reported that the quality of their relationship with patients was similar to their relationships with other patients, 18.8% responded that their quality of relationship was closer than that with other patients, and 2.9% described their relationship as “other.“ Around 79.7% of psychiatrists considered patient suicide predictable, 8.7% said it was not predictable, and 11.6% responded with “I do not know.“

### Serious suicidal attempt

Of the 23 attempted suicide patients, 13 were men (56.5%), 10 (43.5%) were women. 16 (69.6%) were aged less than 35, and the remaining 7 (30.4%) were more than 35 years. Most patients were Saudis 20 (87%). The most common suicide methods were jumping/precipitation (43.5%), cutting/phlebotomy (21.7%), medication overdose/poisoning (8.7%), hanging, fire weapons (4.3%), and drowning (4.3%); three (13%) were not known. In Fig. 1 we demonstrate the diagnoses of the patients who attempted suicide. Slightly more than half 12 (52.2%) of the participants knew their patients for 3 or more months, while 11 (47.8%) knew them for less than 3 months. For 18 participants (78.3%), their last consultation was more than 4 days prior, while for 5 participants, their last consultation was 4 days or less. For most participants (87.0%), their relationship with patients was similar to other patients.

Figure 1 shows the diagnosis distribution among patients who died by suicide or attempted suicide. According to the results, the most common diagnosis among patients was depressive disorder (died by suicide: 43.5% vs. attempted: 34.8%), followed by Schizophrenia (died by suicide: 29% vs. attempted: 30.4%) and substance-related disorder (died by suicide: 17.4% vs. attempted: 8.7%).

**Fig. 2:**
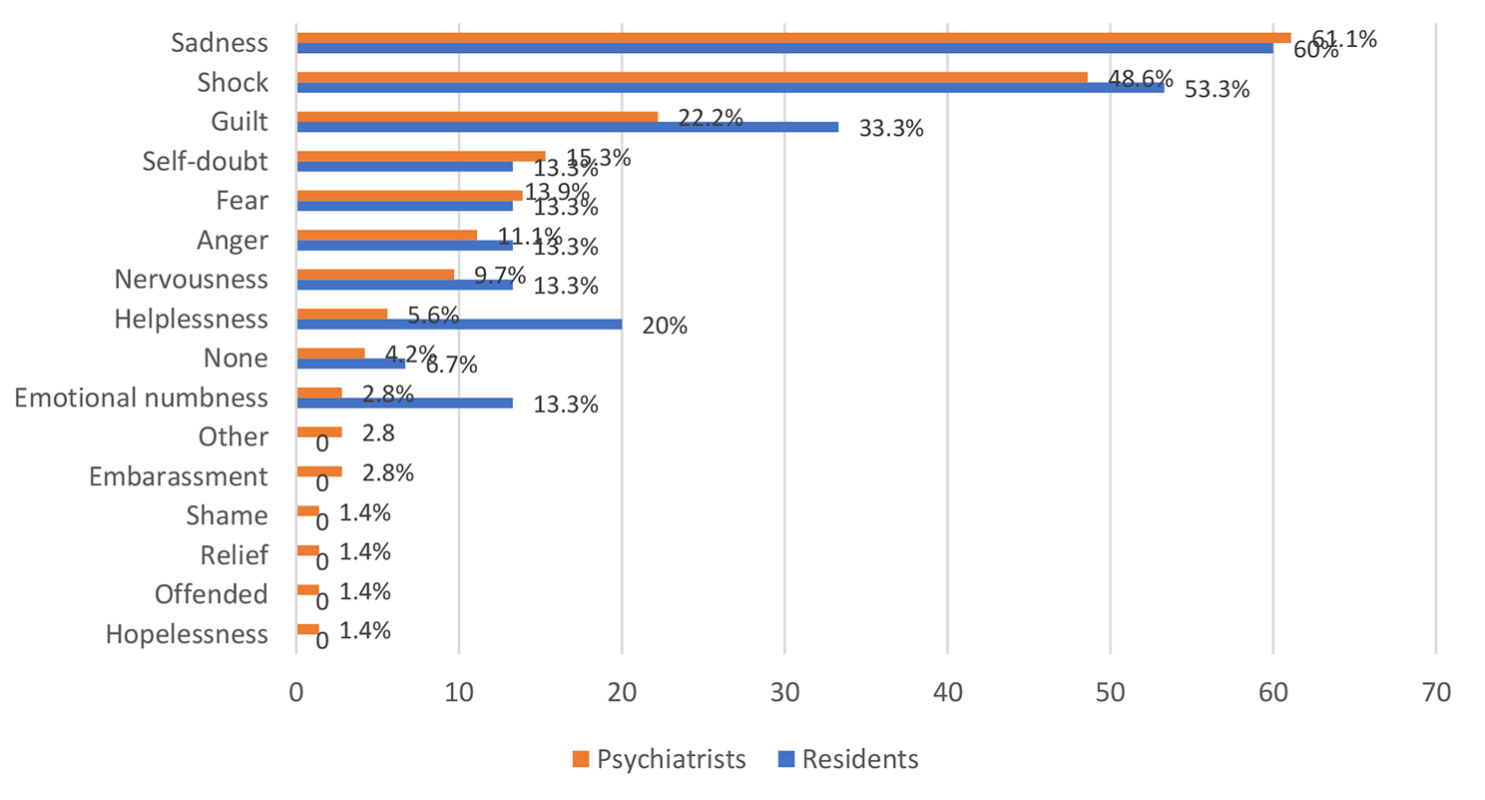
Role of practice in accordance to the emotional reaction after the suicidal event

Figure 2 shows the psychiatrists’ emotional reactions after suicidal events. It can be observed that sadness was the most common reaction of psychiatrists after suicidal events (Psychiatrists: 61.1% vs. residents: 60%), followed by shock (Psychiatrists: 48.6% vs. residents: 53.3%) and guilt (Psychiatrists: 22.2% vs. residents: 33.3%).

**Fig. 3:**
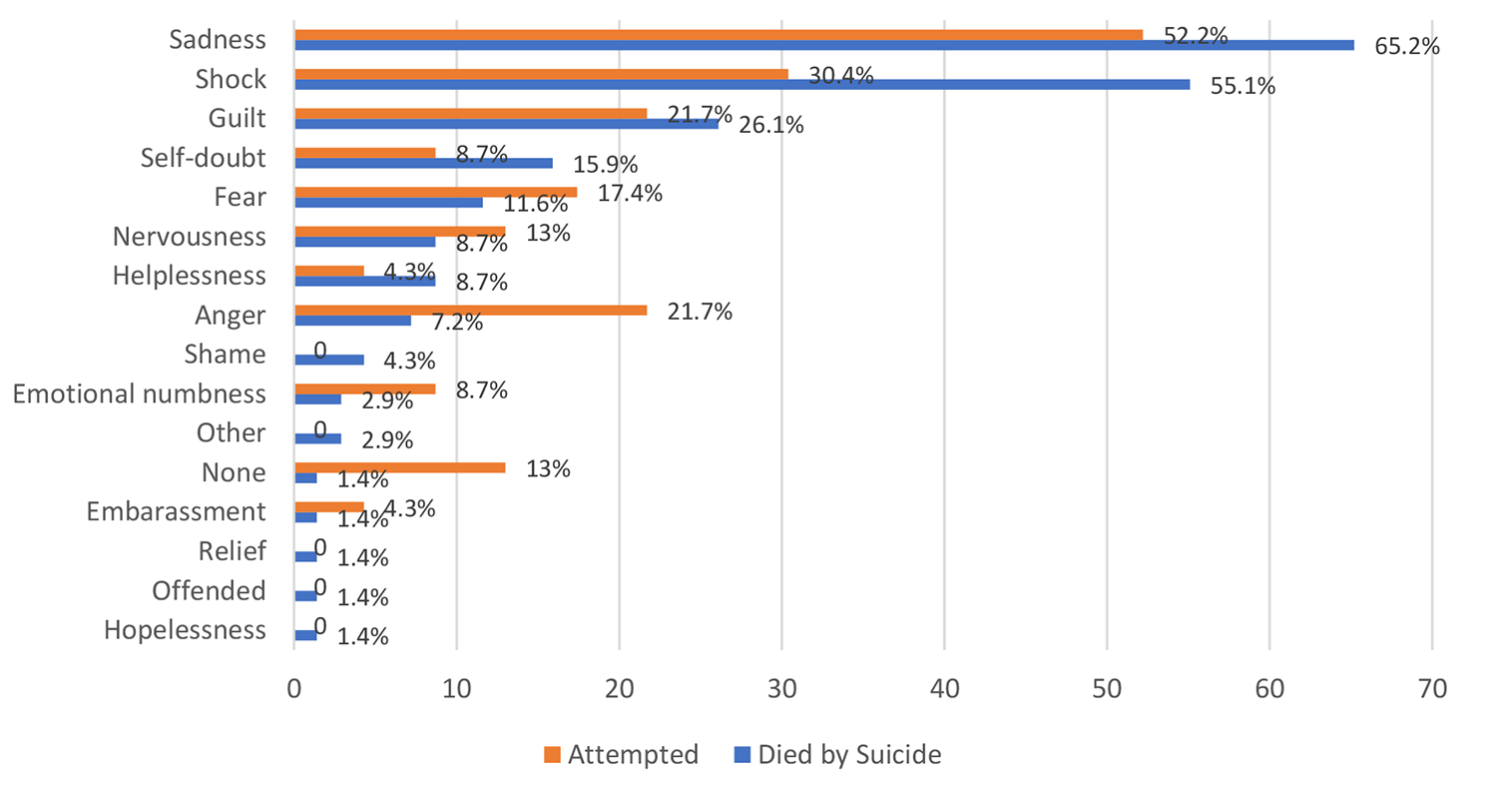
**Type of suicide in accordance to the emotional reaction after the suicidal event**

Figure 3 demonstrates that the most common emotional reaction for psychiatrists after suicidal events was sadness (died by suicide: 65.2% vs. attempted: 52.2%), followed by shock (died by suicide: 55.1% vs. attempted: 30.4%) and guilt (died by suicide: 26.1% vs. attempted: 21.7%).

### Emotional reactions

Psychiatrists responded to a question about their emotional reactions; around 39% reported negative emotions related to completed patient suicide cases, and around 13% reported negative emotions related to patient attempted suicide cases. In addition, the majority reported at least one emotion in relation to both completed and attempted patient suicide cases. The emotions that were most frequently reported were sadness (61.95%), shock (48.91%), and guilt (25%), and these emotions lasted longer with completed patient suicide cases than attempted suicide. In Figs. 2 and 3 most common emotional reactions following suicidal events were demonstrated, comparing psychiatrists to residents and suicide attempts to patients dying by suicide, respectively. Emotional reactions stayed for 1 week to 1 month in most of the cases (47.2%), while others reported less than 1 week (22.2%), 1 month to 3 months (13.9%), 3 months to 6 months (2.8%), and more than 6 months (5.6%). A minority reported no negative emotions (8.3%).

### Impact on professional life

Most respondents 58 (84%), who experienced suicide indicated that suicide impacted their profession. Similarly, many 16 (70%) respondents who experienced a serious attempt were also impacted. 21 (30.4%) of those who experienced patient suicide and 4 (17.4%) of the participants who experienced a serious suicide attempt blamed themselves for the events. In both patient suicide and serious suicide attempts, the most reported professional impacts were increased focus on suicide cues (25.49%), attention to legal aspects (17.15%), and a tendency to hospitalize (12.75%). 66 (71.7%) of the respondents reported that suicide could have been prevented, 22 (30.9%) reported that it could have been prevented by hospitalizing the patient, and 18 (25.35%) indicated that using a different therapeutic approach could have been prevented patient suicide or suicide attempts. The majority 52 (75.4%) of participants who experienced suicide reported that the overall impact of suicidal events in professional practice improved. However, for those participants who faced a serious suicide attempt, only a little more than half 12 (52.2%) reported improvement in the overall impact of suicidal events in the professional practice.

### Impact of event scale-revised

The IES-R score had a low impact on respondents (IES-R total score: M = 12.8, SD = 10.7, range = 0–88). Regarding subscale scores, the highest domain was avoidance (M = 5.42, SD = 5.01, range = 0–24), then intrusion (M = 5.23, SD = 4.35, range = 0–32), and lastly, hyperarousal (M = 2.16, SD = 2.83, range = 0–32), as shown in Table 3. Approximately 10.9% of the total number of respondents who experienced either suicide or serious suicidal attempts of the respondents had symptoms of PTSD (n = 10), with a cut-off of ≥ 25. There were no statistically significant symptoms of PTSD when compared to the type of event (suicide vs. attempt), sex, age, nationality, the role of practice, years in practice, psychiatrist subspecialty, last time seeing the patient, quality of relationship with patients, or characteristics of the patient. However, there was statistical significance among those with anger as an emotional reaction to suicide events (p = 0.029). The three IES-R **domains** revealed some differences in socio-demographic variables. For example, in Table 4, the median scores of older respondents (≥ 40 years) (Z=-2.506; p = 0.012), those with more than 10 years in practice (Z=-2.438; p = 0.015), and non-Saudis (Z=-3.311; p = 0.001) were statistically significantly higher in the intrusion domain. It was also observed that the median score of non-Saudi participants was statistically higher in the avoidance (Z=-2.196; p = 0.028) and hyperarousal domains (Z=-2.645; p = 0.008).

**Table 3:** Descriptive statistics of Impact of Event Scale-Revised (IES-R)

IES-R variables	Mean	SD	Median	Minimum	Maximum
Intrusion score	5.23	4.35	4.00	0	18.0
Avoidance score	5.42	5.01	4.00	0	21.0
Hyperarousal score	2.16	2.83	1.00	0	12.0
Total IES-R score	12.8	10.7	10.0	1	44.0
Symptoms of PTSD	N (%)				
Yes (IES-R ≥ 25)	10 (10.9%)	--	--	--	--
No (IES-R < 25)	82 (89.1%)	--	--	--	--

PTSD – Post Traumatic Stress Disorder

**Table 4:** **Statistical difference of IESR domain and the Socio-demographic Characteristics of psychiatrists** ^a^

Factor	Intrusion Total score (18) Mean (IQR)	Avoidance Total score (21) Mean (IQR)	Hyperarousal Total score (12) Mean (IQR)
**Age group** ^a^			
< 40 years	2.00 (5.00)	4.00 (7.00)	1.00 (3.00)
≥ 40 years	5.00 (5.25)	5.00 (7.00)	1.00 (3.25)
***Z-test; P-value***	-2.506; 0.012 **	***-1.498; 0.134***	***-1.390; 0.165***
**Gender** ^a^			
Male	5.00 (5.00)	4.00 (7.00)	1.00 (3.00)
Female	3.00 (6.00)	5.00 (8.00)	1.00 (6.00)
***Z-test; P-value***	***-0.272; 0.786***	***-0.409; 0.683***	***-0.385; 0.700***
**Nationality** ^a^			
Saudi	3.00 (3.50)	4.00 (6.50)	0.00 (2.00)
Non-Saudi	6.00 (6.00)	5.50 (5.75)	2.00 (2.75)
***Z-test; P-value***	-3.311; 0.001 **	-2.196; 0.028 **	-2.645; 0.008 **
**Role of practice** ^a^			
Resident	5.00 (5.50)	6.00 (6.00)	1.00 (3.00)
Consultant	4.00 (5.00)	4.00 (6.00)	1.00 (3.00)
***Z-test; P-value***	***-0.920; 0.357***	***-0.723; 0.469***	***-0.220; 0.826***
**Years in practice** ^a^			
≤ 10 years	2.50 (4.75)	4.00 (6.75)	1.00 (2.75)
> 10 years	5.00 (5.00)	5.00 (7.50)	1.00 (3.00)
***Z-test; P-value***	-2.438; 0.015 **	***-0.523; 0.601***	***-1.083; 0.279***
**Psychiatrist subspecialty** ^b^			
Child and adolescent	1.00 (1.50)	0.00 (3.75)	0.00 (0.75)
Adult psychiatrist	6.00 (9.00)	6.00 (8.00)	1.00 (3.00)
Geriatric psychiatrist	2.00 (0.00)	3.00 (0.00)	1.00 (0.00)
Psychosomatic medicine	--	--	--
Addiction	4.00 (5.00)	3.00 (7.00)	1.00 (4.50)
None	4.00 (5.00)	5.00 (7.00)	1.00 (3.00)
Others	3.50 (3.25)	4.00 (4.50)	0.50 (1.00)
***H-test; P-value***	***5.733; 0.333***	***7.304; 0.199***	***6.399; 0.269***
**Place of work** ^b^			
Central region	5.50 (5.25)	9.5 (14.75)	2.50 (1.50)
Eastern region	5.00 (3.00)	4.00 (4.00)	1.00 (3.00)
Western region	2.00 (13.0)	4.00 (9.00)	1.00 (6.00)
Southern region	4.00 (3.00)	4.00 (4.75)	0.00 (1.00)
Northern region	6.00 (6.00)	5.00 (6.00)	1.00 (3.00)
***H-test; P-value***	***1.367; 0.850***	***3.061; 0.548***	***4.899; 0.298***
**Workplace** ^b^			
Government	5.00 (5.00)	4.00 (7.00)	1.00 (3.00)
Private	5.00 (14.0)	4.50 (11.5)	1.00 (10.5)
Both	2.50 (2.50)	3.00 (8.25)	0.50 (2.75)
***H-test; P-value***	***2.992; 0.224***	***1.300; 0.522***	***0.750; 0.687***

^a^ P-value has been calculated using Mann Whitney U test

^b^ P-value has been calculated using Kruskal-Wallis test

** Significant at p < 0.05 level

The descriptive statistics of the IES-R and its domains are presented in Table 3. Following the results, the overall median score of IES-R was 10.0 (mean: 12.8), and 10.9% of the respondents had symptoms of PTSD, while 89.1% were normal. The domains’ median intrusion, avoidance, and hyperarousal scores were 4.00, 4.00, and 1.00, respectively.

In Table 4, we measured the differences in IES-R domain scores in relation to the socio-demographic characteristics of psychiatrists. Based on the results, the median scores of those in the older age group (Z=-2.506; p = 0.012), non-Saudis (Z=-3.311; p = 0.001), and those with more than 10 years of practice were significantly higher in the intrusion domain. It was also observed that the median score of non-Saudi participants was statistically higher in the avoidance (Z=-2.196; p = 0.028) and hyperarousal domains (Z=-2.645; p = 0.008). In contrast, the differences in the median scores according to the role in practice, psychiatrist subspecialty, place of work, workplace, and type of suicide were not statistically significant (p > 0.05).

## Discussion

This cross-sectional study assessed the prevalence and impact of patient suicide on psychiatrists and trainees.

### Prevalence

Among the enrolled psychiatrists and trainees, 38.8% experienced patients who died by suicide, which is lower compared to other countries [10–12, 31–35]. There are several possible reasons for this difference: Firstly, Saudi Arabia has a lower overall suicide rate than other countries; secondly, suicide is generally underreported in Saudi Arabia [36]; and thirdly, psychiatrists may not know their patients died by suicide due to lack of communication between different healthcare systems [37]. Additionally, of the participants, 12.9% had patients who had a serious suicide attempt. Compared to other studies, one reported that psychiatrists experienced around 94% of patients’ suicide attempts, and another reported a comparable prevalence of 27.3% [38, 39]. The reason behind the disparity in percentages could be that the first study didn’t specify studying serious suicide attempts.

Furthermore, while looking at the percentage of psychiatrists and psychiatry trainees who experienced complete suicide and serious suicide attempt; for psychiatrists, the percentage was similar to related studies [31, 34, 40]. In contrast, the percentage of psychiatry trainees was slightly lower [31, 33], possibly due to most psychiatry trainees in Saudi Arabia being part of joint programs and rotating between different hospitals, which might limit their knowledge about their patients dying by suicide or undergoing a serious suicide attempt [41]. Considering age and experience, participants with more years of experience and aged 40 years and above were exposed to more completed patient suicides, while participants younger than 40 witnessed more attempted suicide. These findings support the likelihood of the event occurring at least once in every psychiatrist’s career, signifying the importance of psychiatrists being prepared for that inevitable event.

### Emotional reactions

We explored the emotional reactions that psychiatrists may experience following a patient suicide occurrence. To the best of our knowledge, this is one of the few studies to examine the repercussions of attempted and completed patient suicide on psychiatrists, both residents, and consultants. Most respondents reported sadness, shock, and guilt after patient’s suicide. Consistent with some studies, we found characterizing factors, such as age and gender, independent of emotional reactions [42].

Physicians have been noted to be at higher risk of facing occupational-related stress, burnout, and depressive symptoms [43, 44]. Psychiatrists may be at particular risk of developing them [45–47]. The impact of exposure to suicide and suicidal behaviors on psychiatric trainees is hypothesized to be a significant risk factor affecting their mental health [39], which was a similar finding in our study.

### Impact of event scale-revised

In this study, the mean score of the IES-R was 12.8 (SD = 10.7), which is similar to other Swiss [19] and Japanese [48] studies and slightly higher than that of an American study [31] (mean 7.33, SD 11.8). Nearly 11% of participants had alarming clinical symptoms of PTSD, which aligns with other studies [19, 31, 48]. However, compared to older studies, the higher total impact scores, mean avoidance, and intrusion scores are likely because they used the older version of the IES, which reflects the DSM-3 criteria [14, 33, 49]. Interestingly the relationship quality with the patient did not affect the trauma score [50]. We also found that non-Saudi participants (minorities) scored higher on all three subscales (intrusion, avoidance, and hyperarousal). It has been suggested in a previous study that minority psychiatrists may be at higher risk of burnout and depression [46]. Certain socio-demographic factors could contribute to higher levels of intrusion, avoidance, and hyperarousal domains in those who experience suicide events, as noted in the statistical differences in IES-R scores among participants. Additionally, the study underscores the need for mental health organizations to provide adequate support and resources to practitioners who may experience PTSD symptoms or other forms of psychological distress due to their work.

### Professional impact

Most respondents who experienced patient suicide and faced serious suicide attempts noted an impact on their profession. In this study, approximately 91% of the respondents stated that the patient’s suicide led to changes in their clinical practice, with a high percentage mentioning their professional approach has improved, implying remarkable resilience [51].

The majority of participants thought that suicide was preventable. This finding raises the question about psychiatrists’ ability to predict suicide. Despite the general recommendation to utilize suicide risk assessment models in combination with the clinical assessment, their predictive value is low. One meta-analysis reported no significant difference in classifying risk based on individual or multiple risk factors [52]. Another meta-analysis reported that the most significant risk factors related to suicide appeared to have no practical application due to their commonality [53]. While most of our participants did not blame themselves and considered using different therapeutic approaches, the lack of a clear guideline is expected to be challenging, perhaps more so if the experience is repeated. Help in understanding and accepting our limitations in assessment may help alleviate some of the stress that is expected to happen. We also consider the investigation into the effect of multiple suicide experiences on psychiatrists and psychiatric trainees to be of importance.

A recent systematic review concluded that the impact of patient suicide on mental health professionals could be reduced by modifying several factors, including having customizable training to improve risk awareness of suicide. Factors related to the employed institution include nurturing a culture of no blame and gaining knowledge through adverse experiences. In addition, factors related to the type of support offered, individual alterations, and chances of non-formal support were identified [54].

### Limitations

This study has some limitations. First, as noted in similar studies [31, 39], the response rate was low. There are several possible reasons, including time restrictions, as psychiatrists and psychiatric trainees most frequently have busy schedules and demanding duties, especially considering the survey length. Another possible factor is the topic’s delicacy; due to its intense emotional implications, psychiatrists and trainees may be reluctant to participate. Second, the findings may not be broadly generalizable because of the convenient sampling. Third, there is a possibility of recall bias. Given that the data required a retrospective assessment of emotional reactions, major psychiatric disorders’ symptoms, and professional impact, time may have affected the data. Thus, it may not accurately reflect the whole scope of experience following a patient’s suicide. Fourth, this cross-sectional study makes it difficult to discern causality. Finally, although face and content validity methods were used to validate the assessment of the participants’ perceived emotional reactions, these forms of validity may be inadequate. It is subjective and cannot be quantified. Future studies should be longitudinal prospective, have more representative samples, and use well-validated surveys. Despite these limitations, this is the first study, to our knowledge, to examine the prevalence and impact of patients’ suicide and serious suicidal attempts on psychiatrists and psychiatry trainees in Saudi Arabia and Arab and Islamic countries.

## Conclusion

This study confirmed and provided a helpful understanding of the significant emotional and professional effects that patient suicides and serious suicide attempts can have on psychiatrists and psychiatry trainees. The findings of this study highlight the need for further research with deeper analysis and preferably on a regional level, in order to gain more understanding of this important phenomenon and study the usefulness of implementing preparatory and training programs to help trainees and psychiatrists manage the impact of suicide experience.

## Data Availability

The datasets used and analyzed during the current study are available from the corresponding author upon reasonable request.
